# Main challenges to international student mobility in the European arena

**DOI:** 10.1007/s11192-021-04155-y

**Published:** 2021-09-26

**Authors:** Cristina López-Duarte, Jane F. Maley, Marta M. Vidal-Suárez

**Affiliations:** 1 University of Oviedo, Oviedo, Spain; 2 Sabanci University, Istambul, Turkey

**Keywords:** International student mobility, Literature review, Bibliometric, Bologna Process, European Higher Education Area, Challenges to ISM, 120, 123

## Abstract

This study analyses international student mobility (ISM) in Europe since the 1999 Bologna Declaration. International mobility of higher education students is both a driver and a consequence of the Bologna Process and emerges as a relevant issue in a wide range of research areas. This literature review develops a qualitative content analysis of the set of high-performance articles published between 2000 and 2018 and identified through a wide range of bibliometric tools: direct (first generation) citation counts; indirect or accumulated impact; early influence; adjusted impact with respect to year of publication, type of document, and discipline; and alternative metrics that measure interactions in the internet and social media. The content analysis focuses on the pending achievements and main challenges to ISM, among them: attracting non-European students to whole degree programs, the need for actual and further convergence in programs and systems to ensure real compatibility, the impact of HE ISM on the promotion of the European citizenship and consciousness, the sharp imbalance between credit and degree mobility, the need to strengthen the link between ISM and employability, the existing social selectivity in European ISM, the frequent social segregation problems faced by international students.

“Travel is fatal to prejudice, bigotry, and narrow-mindedness, and many of our people need it sorely on these accounts. Broad, wholesome, charitable views of men and things cannot be acquired by vegetating in one little corner of the world.”

Mark Twain

## Introduction

International Student Mobility (ISM)^1^ —the mobility of “individuals who expressly cross borders intending to study” (OECD, 2006)—has been at the forefront of prominence for several decades in Europe. The pace of globalization has increased significantly since the 1980s (Johnson et al., 2006) and it has brought about not only interdependence of national states, but also multiculturalism. Responding to the call for more extensive global competencies for future regional economic sustainability and societal integration, governments and educators in Europe have embraced ISM as an integral part of Higher Education (HE) strategy (Shields, 2016).

The Sorbonne (1998) and Bologna (1999) declarations are the founding statements of the Bologna Process (BP), a milestone labelled as the Big Bang in European HE. It was launched in 1999 and encompassed a wide range of agreements and coordinated policies among European countries in the HE arena that aimed at establishing a completer and more far-reaching Europe. Its objective is to rely on HE to turn the EU into the most competitive economy and knowledge-based society of the twenty-first century (Van Bouwel & Veugelers, 2013). The initial Bologna Declaration (BD) called for six main lines of action to start building the European Higher Education Area (EHEA), being the promotion of (international) mobility one of them. The critical goal of “developing a HE system that shows a world-wide degree of attraction” (EHEA, 1999) relates to the recruitment of students from outside the European area and the promotion of intra-European student mobility flows to such a point that “mobility shall be the hallmark of the EHEA” (EHEA, 2009) —both types of student flows will be considered in this article.

We have come a long way since the Sorbonne, and Bologna Declarations (signed by 4 and 29 countries, respectively) to the current EHEA participated by 48 states, ISM has increased in Europe, and some historic accomplishments have been reached (Shields, 2016; Sin et al., 2017; Souto-Otero et al., 2013; Teichler, 2009, 2012). However, serious challenges persist. This article identifies and analyses the challenges to ISM in the European arena after two decades of the 1999 Bologna Declaration (BD). To this end, we relied on an exhaustive literature review of the most influential articles dealing with this issue published between 2000 and 2018. The review was carried out through a 2-step process: (I) identification of the articles of interest for our research and measurement of their impact on the research field and (II) qualitative content analysis of the articles having the highest impact.

It is organized as follows: the next section introduces the methodology used to select the set of articles to be included in the review and the broad array of bibliometric tools used to measure their impact. Then, a qualitative content analysis of the set of high-impact articles is organized focused on the pending issues and challenges faced to ISM. The article ends with a reflection section that addresses the main challenges in the research field.

## Methodology

We followed the Planning/Conducting/Analysis protocol and the good practices for literature reviews proposed by Pan and López (2004) and Torraco (2005). Our protocol encompassed the following stages—see an extended version in 'Appendix I'.

### Articles identification and selection

Table 1 summarizes the basic features followed in this step. As its main objective was to identify all the articles that potentially addressed our intended issue, no theoretical groundings nor previous content categories were considered ex ante. For the sake of completeness, the search was not limited to educational journals—as shown in Wells (2014), ISM is studied in a broad array of academic fields, among them Education, Migration, and Sociology. The analyzed period is 2000–2018.^2^

**Table 1:** Protocol for the first identification of the articles potentially relevant for our review

Period of study	2000–2018
Type of documents and journals	Full length articles Original research articles & reviews Indexed in SCOPUS and/or the Web of Science (WOS)
Language	English written articles
First identification of potential relevant articles	Keyword search in SCOPUS and the WOS (February/March, 2019) Title/abstract/keywords list: Bologna Mobility + ERASMUS European Higher Educ. Area EHEA

Source: Own elaboration

Through the keyword search, we acquired over 500 potentially relevant articles whose abstracts were read and interpreted by the research team to select the articles to be included in our research. The final dataset brings together 137 articles —the list available upon request.^3^ The growing and diversified expansion of this body of literature is striking: over 70% of the articles were published in the last 6 years and about 50% of the articles were published in journals whose focus is not education, but a broad portfolio of different areas (e.g., economy, engineering, medicine, tourism). The number of scholars involved in this set of articles (over 250 affiliated to more than 160 academic institutions) evinces the interest of the research community in this field. The role of networks is another relevant issue: over half of the articles involve extramural (35%) and/or international (25%) collaboration. Conversely, collaboration with non-academic institutions is exceptionally scarce, as only 4% of the articles in the dataset have been (co)authored by policymakers/implementers, institutional actors (beyond the university area) or firm managers, and less than 20% of the articles rely on externally financially supported research projects.

### Identification of high-impact articles

To assess each article’s impact on the research field we used a wide range of bibliometric tools following the procedure in (self-citation) summarized in Table 2 and relying on the Web of Science (WOS), SCOPUS, and Mendeley databases (information up to July 2019).

**Table 2:** Measurements for the assessment of article’s impact on the research field

Impact	Measurement	Description	Basic references
	Raw citation	First generation of citations	Glänzel and Schoepflin (1999), Kochen (1987)
Direct impact	Citation Per year	Ratio: raw citation counts / years since publication	Tahamtan et al. (2016)
	Early citation	Raw citation counts in the first 2 years after publication	Chakraborty et al. (2014), Guerrero-Bote and Moya-Anegón (2014)
Indirect impact	H index	The citation h-index of the set of papers citing the target one	Fragkiadaki and Evangelidis (2016), Schubert (2009)
Adjusted impact	Field weighted citation impact (FWCI)	Ratio: raw citation counts / expected number of citation counts for similar publications	Kostoff (1997), Vinkler (2003)
	Citation Percentil (CP)	Article’s citation percentile when compared to similar articles	
Altmetrics	Mendeley downloads	Articles downloading and saving activities	Ebrahimy et al. (2016), Erdt et al., (2016), Piwowar (2013), Priem et al. (2012), Weller (2015), Wouters and Costas (2012)

Source: Adapted from self-citation

We firstly carried out a direct (i.e.: first generation) citation analysis. As pointed by Glänzel and Schoepflin (1999) and Kochen (1987), scholars cite in their articles the works that have a direct impact on their research. Citations mean reception, acknowledge, and use by colleagues and place the cited paper into a network context (Schubert, 2009). However, relying exclusively on direct citations, may derive in some biases. As a first step to control for time bias, we split the database into three equally-long sub-periods and identified the articles that compose the field *h-core* in each sub-period; that is, the *h* high-performance articles (with respect to the articles published in the same sub-period) with more than *h* direct citations received (Martinez et al., 2014, p. 1976). Considering per year citation rates and early citation counts are additional means of controlling time-bias. Additionally, early citations allow recognizing the impact of the most recent articles and appreciating innovative research early acknowledged by colleagues (Garner et al., 2014). The Field Weighted Citation Impact (FWCI) and the Citation Percentile (CP) allow controlling biases related to discipline and type of publication.

The measurements described in the previous paragraph asses the articles’ direct impact; that is, they rely on first-generation citation counts. To have a complete picture of each article’s actual impact on the research field, it is advisable to consider its indirect or accumulated influence that relies on further generations of citation and reflects the connection between the target article and the works included in each different generation of citations (Fragkiadaki & Evangelidis, 2016). We relied on each article’s second generation of citations (i.e., citation counts received by each of the articles citing the target one) to calculate each article’s Single Publication h-index (SP h-index). This index is defined by Schubert (2009, p. 560) as “the citation h-index of the set of papers citing it, i.e., not more than h of the papers citing it should receive not less than h citations”. The SP h-index is a robust measurement of an article’s centrality which, in turn, depends on the weight of its citing papers (Schubert, 2009, p.564).

Finally, we analyzed alternative metrics (altmetrics) that rely on the interactions on the internet and the social media platforms. These metrics provide up-to-the-minute information reaching an audience beyond the academic realm (Priem et al., 2012; Wouters & Costas, 2012). Particularly, we relied on the Mendeley social network saving and downloading activity.

These different bibliometric measurements complement each other; therefore, their combined use favors the identification of all high-performance articles, regardless of their age, type, or discipline. Tables 3, 4, 5, and 6 list the set of articles having the highest impact on the research field.

**Table 3:** H Core (2000–2006). Total citation counts and SP H Index

Authors	Year	Raw citation	SP H index
King & Ruiz-Gelices	2003	251	44
Findlay et al	2006	100	28
Papatsiba	2006	52	12
Papatsiba	2005	32	9
Teichler	2001	21	8
Teichler	2003	17	8

Source: Prepared by authors relying on information provided by Scopus

**Table 4:** H Core (2007–2012). Total citation counts and SP H Index

Authors	Year	Raw citation	SP H index
Parey & Waldinger	2011	88	15
Teichler	2009	67	10
Kuhn	2012	54	9
Rivza & Teichler	2007	52	11
Wilson	2011	41	8
Mitchell	2012	29	4
Mechtenberg & Strausz	2008	28	7
Keogh & Russel-Roberts	2009	23	7
Souto-Otero	2008	22	6
Crawford-Camiciottoli	2010	17	4
Teichler	2012	13	3
Goodman et al	2008	12	8

Source: Prepared by authors relying on information by Scopus

**Table 5:** H Core (2013–2018). Total citation counts and SP H Index

Authors	Year	Raw citation	SP H index
Souto-Otero et al	2013	48	7
Mitchell	2015	33	6
Van Bouwel & Veugelers	2013	21	6
Lesjak et al	2015	20	3
Van Mol & Michielsen	2015	19	5
Caruso & De Wit	2015	17	3
Deakin	2014	16	4
Powell & Finger	2013	13	3
Böttcher et al	2016	11	3
Messelink et al	2015	11	2

Source: Prepared by authors relying on information by Scopus

**Table 6:** Articles not included in their respective sub-period h-core that are among the top-25 relying on per-year, or early, or adjusted, or altmetrics impact

Authors	Year	Per-year	Early 2	FWCI	CP	Mendeley
Almeida et al	2016	–	4	2,3	–	42
Beerkens et al	2016	4,0	6	–	–	53
Borghetti & Beaven	2017	–	–	2,7	–	
Bótas & Huisman	2013	–	–	–	–	57
Brooks	2018	8,0	–	4,8	–	50
Çiftçi & Karaman	2018			2,5	–	
Dvir & Yemini	2017	4,7	7	5,1	93	
França et al	2018	6,0	–	2,4	–	
Golubeva et al	2018	–	–	1,9	–	
Jacobone & Moro	2015	2,9	–	2,1	–	105
Llurda et al	2016	–	4	–	–	
Pásztor	2015	–	4	–	82	
Savenkova & Svyrydenko	2018	4,0	–	–	–	
Shields	2016	–	5	2,0	–	38
Sin et al	2017	–	4	1,8	–	
Tommasini et al	2017	–	–	–	–	61
Van Mol C	2018	4,0	–	–	–	
Wihlborg & Friberg	2016	–	–	7,5	93	48

Source: Prepared by authors relying on information by Scopus and Mendeley

### Information extraction and building of a codebook

A second database was built encompassing the high-performance articles identified in the previous stage. For each article, some bibliometric data was gathered, among other, the keywords placed by the authors and/or by the SCOPUS or WOS databases. In addition, key information was extracted and coded by the research team: article’s main objective, geographic focus and scope, discipline approach, empirical analysis (if any) and its features, main results, and conclusions.

Relying on the articles’ keywords a codebook was built containing the main descriptors within the field. A total of 154 different words and compound forms were identified in the first round of analysis. The research team grouped these words/forms thematically giving rise to a former list of descriptors that was reviewed through an iterative process until arising to a significant limited list of descriptors in terms of content and frequency. These were lastly integrated in 3 main categories an different subcategories: (I) ISM objectives —different subcategories were identified dealing with the different stakeholders involved in the ISM: students, scholars, HE institutions, countries/societies, and supranational institutions (i.e., the EU). (II) Achievement degree of these different purposes. (III) Policies, programs, and tools favoring ISM and the accomplishment of the above mentioned objectives.

### Qualitative content analysis

Using this codebook as a framework and guiding structure, the research team carried out a qualitative analysis of the extracted information and the articles’ content focused on the main challenges and pending achievements faced by ISM in the European context.

## Qualitative content analysis: challenges to ISM

**Fig. 1:**
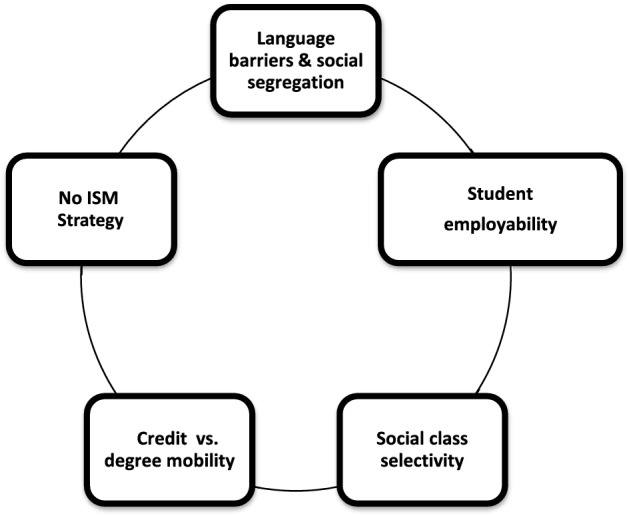
The challenges of ISM in the European arena

There still exist many challenges to ISM despite the best endeavors of policymakers and HE specialists. Five critical challenges identified are illustrated in Fig. 1: student employability, social class segregation, credit mobility, the strategic intent of ISM and language barriers. These challenges will now be discussed.

### Strategic intent of ISM

As a first step, the EHEA has not been entirely successful in achieving the strategic objective of increasing inward ISM for whole degree programs from non-European countries (Rivza & Teichler, 2007; Shields, 2016; Teichler, 2012). As mentioned earlier, this was an explicit objective in the BD. However, a substantial variation among countries exists with a clear division between Western and Central and Eastern European countries (Shields, 2016). The latter facing more severe difficulties in attracting students from non-EHEA countries. The trends indicate that ISM and outward student flows from some emerging powerhouses (i.e., China, India) are increasing exponentially (Rivza & Teichler, 2007). This scenario provides a strategic opportunity for European HE institutions to attract students beyond European borders. Inward European vertical mobility is often criticized for benefiting the financial elites, contributing to brain drain in the students’ home countries; maintaining host countries’ strategic leading role and influence over the home developing ones (sometimes former colonies), and actively calling for adaptation (Franca et al., 2018; Teichler, 2009; Wells, 2014). This vertical degree-mobility is often viewed by students as the first step towards future migration (Rivza & Teichler, 2007; Sin et al., 2017; Wells, 2014). A fact which raises the potential contradiction of international students as desired (due to economic and brain-gain reasons) and unwanted (due to migrations controls and politics) (King & Raghuram, 2013) and the need to tackle within the BP the geopolitical challenges faced by Europe. Furthermore, the interest on attracting non-EHEA students may also challenge the cooperation paradigm among European HE institutions and give rise to increasing competition for quality and market share (Pazstor, 2015; Teichler, 2003, 2009; Van Bouwel & Veugelers, 2013).

In addition, the strategies and policies between European HE systems (including those related to ISM) bear vast differences. One of the more pending issues is that many nations are still preoccupied with their national strategy, which is often not aligned with the spirit of the BP (Brooks, 2018). What is more, these countries typically lack a clear strategy to change this situation. The actual convergence of structures and compatibility of systems among the European countries still needs further development. It seems that the convergence discourse placed in the BD is far ahead of its practical implementation; and this affects different action lines, among them, the promotion and management of ISM. This is a crucial issue not only hampering the development of a world-wide attractive HE system, but even hindering the credit or temporary mobility (i.e. the most popular mobility type in the European area, as explained in the following paragraphs). Furthermore, some basic issues such as, for instance, the academic calendar, the language requirements, or the academic prerequisites for accepting (short or degree) mobile students widely differ not only among EHEA countries, but even among universities within the same country.^4^ These differences hinder the comparison and integration of academic programs, as well as the combination of academic periods in different universities. Therefore, administrative convergence is also needed to foster both short-term/credit and degree mobility intra and inter-EHEA.

Within the European arena, ISM goes far beyond the education/qualification context as it is a matter of “deepening relations with other European countries” (EHEA, 1999). Contributing to enrich European citizenship, the stock of shared values and the feeling of belonging to a common social and cultural space is a crucial goal of the EHEA founding statement. In this context, ISM is both a driver and a consequence of the EHEA building process. Although the BP has gone some way to building a politically integrated Europe, the recent fallout from BREXIT may throw doubt on this claim, and it is challenging to specify the extent of success in this case.

### Credit versus degree mobility

In contrast to other world regions, credit (also known as temporary or exchange) mobility (i.e. a short-term period of study that relies on exchange agreements between institutions and means no payment of tuition fees at the host institution) is the prevalent form of ISM in the European arena; with degree mobility playing a much-limited role and increasing at a lower rate. The European Region Action Scheme for the Mobility of University Students (ERASMUS) formerly launched in 1987 and focused in credit mobility has become the most popular scheme for student mobility at the European level, the flagship of the EU educational programs, and one of the most successful single components of EU policy (Papatsiba, 2006; Teichler, 2001). Attenuating the huge imbalance between credit and degree mobility remains an irksome challenge to the EHEA.

Even when the focus is placed on intra-European credit mobility, some challenges are identified: regardless of its overall growing trend for both studies and placement (Shields, 2016; Sin et al., 2017; Souto-Otero et al., 2013; Teichler, 2009, 2012), the benchmark of 20% by 2020 placed in Leuven (EHEA, 2009) is not affordable. In addition, country members show quite different paths, speeds, and achievements regarding ISM inward and outward flows and at the present time, a scarce 10% of the EHEA countries encompass HE institutions that include compulsory mobility periods as part of some study programs. The above mentioned lack of actual convergence arises as a key barrier to credit mobility, as the recognition of studied modules, rigid course organization, credits transfer, non-consensual competency evaluation and, even, schedule clashes are among the most frequent challenges and barriers faced by mobile students (Beerkens et al., 2016; Papatsiba, 2006; Powell & Finger, 2013; Sin et al., 2017; Souto-Otero, 2008; Souto-Otero et al., 2013; Teichler, 2003, 2009; Tommasini et al., 2017). A second one is the social class selectivity that we address in the following point.

### Social class selectivity

Selectivity for ISM relies to a substantial degree on the student's ability to pay a significant portion of the cost of the ISM sojourn typically. As a consequence, a large proportion of students who complete the ISM experience come from families with an above-average economic status and higher educated parents (Beerkens et al., 2016; Caruso & De Wit, 2015; Findlay et al., 2006; Pásztor, 2015; Powell & Finger, 2013; Souto-Otero, 2008; Souto-Otero et al., 2013). Thus, a selection approach that is mainly dependent on a student's funding raises the question of to what level social inequality affects ISM involvement. Inequality makes ISM a dream for students from lower socio-economic classes, and it does not even come on the radar for most students from underprivileged backgrounds (Choudaha, 2017; Christie, 2007).

The root of inequality in ISM often stems from inadequate funding (Choudaha, 2017), as grants and sponsorship do not nearly meet the needs of many students (Findlay, 2011). Despite some success in reducing funding barriers (Cairns, 2019), social selectivity continues to obstruct ISM (Teichler, 2012). Consequently, ISM still reaches only a minority of HE students despite the goals of the Bologna processes to make ISM available across all social classes (EHEA, 2009). This is the case even in exchange and credit mobility programs, and social inequality in Erasmus policy debate appears to be an issue that frequently gets overlooked.

In addition to financial issues, another concern is that low socio-economic students (and their families) often do not even get to hear about ISM and often lack total awareness of the benefits and value of ISM (Teichler, 2017) —as before said, parental education/background is another key driver of social selectivity. Social inequality also impacts on the student awareness of the ISM scheme, which, in turn, affects their motivation to study abroad (Bryła & Ciabiada, 2014). In other words, social inequality impacts knowledge transfer regarding ISM. Consequently, in addition to the other impediments, students get no encouragement from home to participate in ISM. The lack of support is particularly pertinent in migrant, second-generation students and tends to negate the pull factors for ISM.

Cairns (2019) makes a compelling case that to understand the issue of social inclusion in ISM fully, it is necessary to examine the financial governance of the program and student grant allocation process in greater detail. Findlay (2011) takes a closer look at what shapes the financial interests of those who organize the ISM program and considers that ISM is one of the social fields where societal inequalities are unwittingly recycled. Certainly, ISM students can expect to receive higher wage growth after graduation (Kratz & Netz, 2018); and those that are excluded receive on average -lower wages. Consequently, ISM may play a part in the reproduction of social class (Findlay, 2011). Perhaps the most crucial point in addressing the fundamental and future challenges in social inclusion is first “addressing the issue that students who do participate in Erasmus appear to have limited awareness of their relatively privileged position” (Cairns, 2019, 145). There is still much learn in all aspects as to what may be preventing HE students from lower socio-economic classes engaging in ISM. One thing that evidence is very clear about is that ISM remains elitist, and efforts to change the status quo have not been effective. A lot remains to be done to fix this problem.

### Student employability

Employability and finding a job after graduation is still a fear for many students throughout the EU, and it subsequently impacts upon their ISM choice (Nilsson & Ripmeester, 2016). Improving their professional development is among the key objectives pursued by students when deciding to study abroad (Jacobone & Moro, 2015; Keogh & Russel-Roberts, 2009; Papatsiba, 2005, 2006; Souto-Otero et al., 2013). The argument supporting this professional objective is that global competitors surpass the capacities of each European nation taken separately (Papatsiba, 2005). ISM is a source of higher academic qualification and cross-cultural competencies that increases students’ opportunities to access the (international) labor market, face better employment prospects, and work in multicultural contexts. This employability focus is particularly relevant in countries that face high graduate unemployment rates (Sin et al., 2017), as well as in ISM for placement (Deakin, 2014). Furthermore, in the case of vertical mobility (moving towards economically more advanced and academically superior systems), ISM plays a role as a possible first step towards future migration (Rivza & Teichler, 2007; Wells, 2014).

Despite some headway towards making ISM graduates more employable and positive moves in rectifying transition problems between HE and the labor market, several authors have pointed out that several challenges remain (Cairns et al., 2018; European Commission, 2014; Roy et al., 2019; Soares & Mosquera, 2019; Wilton, 2011). One of the first challenges in helping ISM students acquire employability skills may lie at the heart of academic resistance and prejudices to the concept of HE for employability. For example, the employability in ISM schema pushed by EU governments is argued by Altbach and Knight (2007) to be at odds with the humanist concept of education as ‘something for the public good.’ For some academics, the drive for employability skills as part of HE curriculum is too embedded within the neoliberal agenda in HE and crushes the humanist HE aims of education (Collini, 2012).

A second challenge is the need to cultivate the linkage and build stronger collaborations between HE and industry (Cairns et al., 2018). The collaboration between ISM organizers and the workplace could enhance skills desired by contemporary employers, and thus, to enhance employability for ISM graduates. The UK’s Sussex University have developed a successful prototype for improving university-industry networks (King & Ruiz-Gelices, 2003), and this model could be rolled out across the EU.

A third concern is that ISM has, in general, come under criticism in terms of unrelated and useless course content that will not make a practical contribution to helping students find work when they graduate (Messelink et al., 2015). While individual sector-specific technical skills have typically been part of HE curriculum in many parts of the EU, ‘soft’ skill development for employability has been neglected. Soft skills include attitudes, behaviours, and the ability to work in teams (Muir, 2004). Soft skills are critical enablers of graduate ability to function effectively in the modern workplace, and their development is now considered integral to HE (Jackson, 2015), and typically ranks the highest in recruiter preference (Jones et al., 2017).

A fourth challenge relates to a more significant endorsement of ISM for student employability. The ISM needs to retain a student perception of exclusiveness and distinctiveness (Souto-Otero, 2008; Teichler, 2009), while at the same time, extend. These objectives are not necessarily mutually exclusive (Soares & Mosquera, 2019). Perhaps, we can look to the work-integrated learning (WIL) schema to support the ISM—employability liaison (King & Ruiz-Gelices, 2003). The WIL program is considered instrumental to graduate job-readiness (Wilton, 2011), and improves explicitly soft skills (Jackson, 2015). The evidence is clear that student employability in ISM is a necessity, not a choice (Pollock, 2014). As one of the critical objectives of Bologna, the work competency enhancement gap between HE and subsequent employment is far from closed and has some way to go before it can rise to meet future commercial and technical challenges.

### Language barriers, cultural challenges, and social segregation

Language barriers are extensively acknowledged as a critical barrier to intra-European ISM (i.e., Beerkens et al., 2016; Goodman et al., 2008; Keogh & Russel-Roberts, 2009; Powell & Finger, 2013; Souto-Otero et al., 2013), regardless of the increasing use of English as the *lingua franca* (Borghetti & Beaven, 2017; Crawford-Camiciottoli, 2010; Tommasini et al., 2017; Wells, 2014). Taking lectures in a language different from the local one fosters the creation of language-based clusters that keep international students away from locals. Other organizational factors usually exacerbate the social segregation of international students and push students to get involved in socialization processes in the host country that rely on co-national and international (rather than local) student networks (Van Mol & Michielsen, 2015). Among others: (I) the use of bilateral agreements that lead to a concentration of compatriots in the same host cities/institutions; (II) the type of courses taken by mobile students and their accommodation options —most often, credit students take a mix of courses from different degrees and academic years and are accommodated with other international students,^5^ and even (III) some mobile credit students using ISM as a grand *fiesta* —with no real academic ambition.

ISM positively impacts the students’ cultural awareness, intelligence, sensibility, empathy, and adaptability and helps them to develop their intercultural competences, cross-cultural communication skills, and global mindedness, among other benefits —see Roy et al. (2019) for an exhaustive review. ISM is a socialization process that influences people’s capacities to live (not only work) across national and cultural borders and develop their identity and a sense of belonging (Wilson, 2011). The achievement of these cultural outcomes depends, amongst others, on the mobility duration and the students’ social interaction during the mobility (Roy et al., 2019). However, short-term or credit mobility (the most frequent option within the EHEA) provides a limited opportunity for the above-mentioned cultural achievements. Additionally, the already mentioned language and organizational factors usually hinder interaction with locals, once again, preventing students from cultural achievements. Furthermore, language and cultural differences have traditionally hindered the actual convergence of educational systems in the EHEA (Van Bouwel & Veugelers, 2013) and have helped create factions among students.

Coordinating student flows at a national level, developing academic organization and accommodation solutions that foster interaction with locals and multicultural environments, and developing formal and non-formal intercultural interventions that foster the students' intercultural competencies (Almeida et al., 2016; Messelink et al., 2015) would somehow alleviate these problems.

### The virtual mobility challenge

International virtual mobility deals with virtual learning supported by ICT that involves cross-border and cross-cultural collaboration and provides students the opportunity to get the same rewards as traditional physical mobility without having to travel (Being Mobile, 2010). It can facilitate overcoming some of the above-mentioned drawbacks of traditional mobility programs (for instance, the social selectivity problem rooted on financial restraints) and increasing students’ accessibility to mobility projects (Maček & Ritonija, 2016). Despite its potential advantages and the implementation of the Erasmus + virtual exchange program in 2018, virtual mobility is still an option to be further explored and implemented in the EHEA, as shown in some pioneering projects —see, for instance, the projects for engineering and nursing students in Menéndez-Ferreira et al. (2017) and Wihlborg and Friberg (2016), respectively; the study on mobility coordinators’ opinion and attitudes to virtual mobility in Abramuszkinová-Pavlíková (2014), or the analysis of the feasibility of virtual mobility programs for master degree students in Ruiz-Corbella et al. (2014).

The crisis caused by the COVID-19 virus pushed many EHEA universities to engage in virtual learning activities, among them international mobility activities, without having planned them in advance. The disruption caused by the crisis resulted in many HE international mobility programs abruptly switching to virtual programs. Moreover, it appears that crisis has propelled and advanced the development of EHEA virtual mobility. Perhaps, international virtual mobility has finally come into its own, following several years of hesitation, involving planification, organization, and deep analysis.

## Final remarks

ISM is vitally crucial for European development and sustainability in terms of its ability to act as a capability enabler that will serve Europe in the future where knowledge and global awareness will together be essential capabilities (Gonzalez et al., 2011). A broad array of stakeholders exist whose objectives and expectations related to ISM may differ substantially —students, academics and HE institutions, policymakers, countries, and supranational institutions. Consequently, the dimensions of HE in the European arena have become confusing and political (Papatsiba, 2005, 2006). Although achievements are undeniable, there remain areas for significant improvement in the political, strategic, professional, economic, and social domains.

## Research agenda

As our qualitative content analysis of the set of selected articles has been focused on the main challenges and pending achievements faced by ISM in the European context, all the issues raised in that section deserve further attention and additional research: the need to develop the policies, tools, and programs that allow increasing inward ISM for whole degree programs from non-European countries; the analysis of existing differences among EHEA countries regarding this issue (and that of credit mobility); the actual convergence of structures and compatibility of systems among EHEA countries; the BREXIT challenge to the EHEA’s goal of promoting the European citizenship; the social class selectivity problem of international student (credit) mobility; the international mobile students employability; their social segregation due to, among others, cultural issues; and the virtual mobility challenge.

The analysis of country differences in their attraction of non-EHEA students and promotion of intra-EHEA credit mobile students should be linked with the economic and political objectives that ISM can yield at the country and supranational level, among them^6^: (I) the development of the European labour market; (II) the enhancement of the economic cooperation among European partners and the increase of the European international competitiveness; (III) the increase of current and future revenues for host countries —ISM programs are an essential contributor to the tourism and HE industries—; (IV) the development of skilled workforce (brain gain) through the retention of international students after graduation —a particularly relevant issue in contexts of decreasing and ageing populations, as it is the case of many Western UE countries—; (V) the promotion and development of the already mentioned European citizenship and consciousness that, in turn, enhance knowledge of the historical and cultural aspects of Europe, facilitate awareness of common sociopolitical issues, support the European integration, and enable cultural, societal, and political international understanding.

Furthermore, the national-level analysis should be enriched with an in-depth study of the role played by the universities’ traits, among them, their excellence and reputation (Beine et al., 2014; Delgado Marquez et al., 2013; Schnepf et al., 2020). ISM facilitates competition among universities to attract students giving rise to an increase in their quality, international prestige, and reputational capital (Mechtenberg & Strausz, 2008; Souto-Otero et al., 2013; Van Bouwel & Veugelers, 2013). Incoming international students are, in turn, a means to diversify the sources of financial resources and to increase income (Pásztor, 2015; Rizva & Tecihler, 2007).

ISM is just one form of internationalization in HE (Teichler, 2009). However, other forms of ISM exist with challenges that also deserve further attention and study as, for instance, the development of transnational study programs and dual degrees jointly offered by international partners, the teaching staff mobility, and the internationalization at home or virtual ISM (Rivza & Teichler, 2007; Teichler, 2001; Wells, 2014; Wihlborg & Friberg, 2016). Research on the international virtual mobility issue is particularly urgent. As before said, the COVID-19 crisis has pushed many universities to engage in virtual mobility program without planification, adapted programs, nor previous analysis of its potential advantages and shortcomings. Research is needed, among other issues, on these programs actual feasibility; the students’ attitudes to them and their valuation of the social, academic and employability/professional opportunities that a virtual program can offer, the students’ actual willingness to engage in such virtual programs, and the teachers’ and programs coordinators’ positioning about them.

### The need for theoretical advancements

As already pointed by Roy et al. (2019) in their valuable review on the outcomes of ISM, there is a need for studies that draw on theoretical foundations to explain ISM. We fully share their proposal relative to the use of social learning, experimental learning, person-situation, social-psychology, and intercultural relation theories to further explore ISM outcomes, the groups of students that benefit the most from a mobility experience, and the boundary conditions that may influence the effectiveness of international mobility programs —see Roy et al. (2019) for an exhaustive review. Theories borrowed from the organizational field can also play a role in helping analyze ISM traits, results, and design, among others, the Paradox Theory and the Co-evolution Theory.

The Paradox Theory is traditionally known as an organizational theory; however, it draws from and can be used in related disciplines (Bednarek et al., 2021) and it can be useful to analyze individuals and their social interactions (Waldman et al., 2019). A paradox deals with “contradictory yet interrelated elements that exist simultaneously and persist over time” (Smith & Lewis, 2011: 382), pushing individuals to address competing demands simultaneously and to engage and accommodate tensions, rather than resolve them (Smith & Tracey, 2016). Recently, this approach has been used within the educational field to explore the university–community partnerships (Strier, 2014), understand and manage change in medical education (Gordon & Cleland, 2021), teacher evaluation processes (Paige, 2013), and as a tool to foster the development of students’ capabilities in Management education (Knight & Paroutis, 2017), among others. It can be also a suitable lens to explore the multiple tensions linked to ISM (such us social-academic objectives, easiness to pass-curriculum distinction, comfort zone-multiculturalism engagement, home university demands-host university requirements, etc.). As pointed by Lewis (2000) and Smith and Lewis (2011), the more global, dynamic, and competitive the environment becomes, the more intense the contradictory demands are. The fast emergence of virtual mobility as an additional alternative to ISM, has sharply increased these tensions by adding the isolation-mobility paradox (Daniel et al., 2018).

The Co-evolution theory can be useful to explore the interplay between the activities by universities and the evolution of their institutional environment. This organizational theory propose that firms co-evolve with their environment and play a role as change agents, rather than being mere actors that adapt their activities and strategies to the existing institutional environment and/or select the specific environment in which they can better perform their activities (Baum & Singh, 1994; Lewin & Volberda, 1999; Volberda & Lewin, 2003). In the ISM field, a driving force of the institutional evolutionary process is the way in which universities design their programs and perform their activities. This is particularly relevant when a crisis takes place and uncertain and complex situations must be faced (e.g. the COVID 19 crisis and the sudden turn to virtual mobility of traditional international mobile students). In these cases, the universities actions and decisions do not only mean an adjustment to the existing institutional environment, but affect change in the institutions (Cantwell et al., 2010).

### Main limitations

The following issues arise as the main limitations of this review article: as it has been pointed in different parts in this article, different realities exist within the EHEA, as different countries show diverse paths, speeds and, even, commitment to ISM. As the review process has been limited to articles written in English, contributions in other languages that may analyze specific countries’ differentiated realities have not been considered. This is for sure an area of interest for future research. Furthermore, a comparison of the main challenges faced by ISM within and outside the EHEA is of interest.

As the review is focused on high impact articles, methodological restrictions impede to consider articles published after 2018. In addition, contributions by articles that do not have a high impact on the research field have not been considered. Considering both, recent and (by the moment) low impact articles would be a way to extend this review.

In addition, the pieces of research included in our dataset are exclusively full-length articles published in journals indexed in the Scopus or WOS databases. Other contributions, as articles published in non-indexed journals, books, book chapters, research notes, or conference proceedings have not been considered. Considering these outlets is another avenue for extending this review; just for instance, conference proceedings usually encompass the first version of pieces of research that show fresh and challenging approaches to the analyzed issue.

Finally, our content analysis is a qualitative one; that is, it relies on the analysis and interpretation of the articles’ contents carried out by the research team. Although this methodology allows for a deep and thorough analysis of the articles’ contents, it also means some degree of subjectivity. An additional way to extend this research would be to carry out a similar review relying on tools for content analysis that allow for a higher degree of objectivity.

## Protocol for articles identification, selection, and analysis.

### Stage A. First identification of the articles potentially relevant for the research

- A.1. Focus of the issue: International student mobility (ISM) in Europe since the Bologna Declaration (BD) in 1999.
- A.2. Period of study: 2000 to 2018. The starting year is the first one after the signature of the BD. The end of the period is placed in 2018 due to methodological restrictions, as citation processes require a minimum period of time (i.e.: the citing paper must be not only developed and submitted to the journal, but also reviewed, accepted, and published).
- A.3. Type of pieces of research: full length articles (original pieces of research and reviews) published in academic indexed journals. These pieces of research are reviewed by academic peers before acceptance to be published in journals whose prestige relies, among other issues, on citation processes. These practices mean recognition by the academia (Podsakoff et al., 2005, p. 473). Consequently, this kind of articles can be considered as “certified knowledge” (Ramos-Rodríguez & Ruíz-Navarro, 2004, p. 982).
- A.4. Databases: SCOPUS and the Web of Science (WOS). These two databases have been traditionally considered “the golden standard” for journal articles coverage and bibliometric data (Harzing & Alakangas, 2016; Harzing & van der Wal, 2008).
- A.5. Language: English, as this is the “lingua franca” in international higher education (Berns, 2009; Jenkins, 2013; Smit, 2010) and “the world language of academia… used by the research community… (including) non-native scientists and scholars” (Mauranen et al., 2010, pp. 183–184).
- A.6. Keyword search: the first identification of potential relevant articles was carried out through a keyword search performed in February/March, 2019 in both Scopus and the Web of Science (WOS), looking for all the articles including in their title, abstract, or keywords list the word “mobility” combined with any of the following ones: “Bologna”, “ERASMUS”, “European Higher Education Area”, “EHEA”.

The wide scope of the keyword search favored the identification of a high number of articles coming from non-educational journals. Once duplicates were removed, we got a former set of more than 500 articles.

### Stage B. Selecting the articles

The abstracts of these articles (full articles when necessary) were read and interpreted by the research team to decide whether each article actually dealt with the intended issue stated in A.1: International student mobility in Europe. All the articles dealing with this issue, regardless of their focus, theoretical approach (if any), empirical content (if any), descriptive nature, discipline, or field of research were included in our database.

At least two different researchers read each single article/abstract and made an independent decision on it. Discrepancies were solved through a review process by all the team members and a subsequent group debate.

We arose to a final dataset of 137 articles. The removal of more than 70% of the identified articles was the consequence of the wide scope of the keyword search.

### Stage C. Identifying the articles having the highest impact on the research field

A wide range of bibliometric tools was used to identify the articles having the highest impact on the research field: raw or direct citations counts (i.e. first generation of citations), h-cores for different time-frames, per-year citation rates, early citation counts, field-weighted adjusted impacts, citation percentiles, accumulated impact (i.e. indirect citation counts relying on the second generation of citations), and alternative metrics (e.g. Mendeley downloads).

Information about all these metrics was collected for each of the 137 articles in the database. A high-performance set of articles was identified that encompasses: all the articles included in their respective time-frame h core plus all the articles that are not included in these h cores, but are ranked amongst the top 25 relying on at least one of the following metrics: per year citation rates, early citation counts, field-weighted citation impacts, citation percentiles, or alternative metrics (i.e. Mendeley downloads).

### Stage D. Information extraction and building of the codebook

A second database was built encompassing the high-performance articles identified in the stage C of this protocol. For each article, some bibliometric data was gathered, among other, the keywords placed by the authors and/or by the SCOPUS or WOS databases. In addition, key information was extracted and coded by the research team: article’s main objective, geographic focus (e.g. it is was focused in a specific country or group of countries), discipline approach (i.e. if the article was focused on an specific higher education discipline like, for instance, nursing, business or engineering), empirical analysis (if any), main results, and main conclusions.

Relying on the articles’ keywords a codebook was built containing the main descriptors within the field. A total of 154 different words and compound forms were identified in the first round of analysis. Information about each word/form frequency was also gathered. The words/forms “student”, “mobility”, and “higher education” were removed, as they compose the focus of our intended issue and they appeared in almost all the articles in the dataset. Some words that dealt with specific methodological issues (e.g. regression analysis, interpretative phenomenological analysis), specific disciplines (e.g. nurse, engineering), and specific countries (e.g. UK, Spain, France) were also removed.

The research team grouped the remaining words/forms thematically giving rise to descriptors. The initial list of descriptors was reviewed through an iterative process eliminating some categories and merging similar ones. We finally arose to a significant limited list of descriptors in terms of content and frequency that were lastly integrated in 3 main categories an different subcategories: (I) Mobility purposes (different subcategories were identified relying on the different stakeholders involved in the ISM: students, scholars and HE institutions, countries/societies, and supranational institutions (i.e., the EU). (II) Policies, programs, and tools favoring ISM or fostering the achievement of the above mentioned objectives (III) Achievements and results (once again, differentiating among student or individual attainments, feats at institutional or university level, and achievements at national and supranational levels).

### Stage E. Qualitative content analysis

Using the codebook as a framework, the research team carried out a qualitative content analysis of the articles’ content and the information extracted in the stage D of this protocol. The analysis focuses on the pending achievements and challenges to ISM in the European arena.

## Footnotes

1 See Wells (2014) for an exhaustive review of the different academic fields that study ISM and the broad array of definitions and concepts associated with this phenomenon.

2 2000 was chosen as starting point as it is the first year following the signature of the BD. The choice of 2018 as end of the analyzed period is due to methodological restrictions related to the citation process (see the *Identification of high-impact articles* section and 'Appendix I').

3 The removal of more than 70% of the identified articles is the consequence of the broad scope of the keyword search —the narrower the search scope, the higher the risk of losing relevant pieces of research. ISM is not only one of six basic lines of action in the BD, but it is tightly affected by the remaining five (i.e.: the adoption of a system of legible and comparable degrees, the introduction of a two-cycle system of study programs (undergraduate and graduate), the establishment of a system of credits to measure students’ achievements, promotion of cooperation in quality assurance, and promotion of the European dimensions in HE). This means that nearly all the articles that deal with the Bologna process include a reference to international student mobility, even when they are not focused on this topic or they do not further analyze any issue related to it. Basic bibliometric information was gathered and codified for each selected article (i.e. authors, journal, year, title, and keywords, among others).

4 E.g.: the starting point of the academic year differs in over 2 months among EHEA countries and universities; some EHEA universities concentrate holydays in a 8–10 weeks period after the Spring semester, while others split them in two different 4–5 weeks periods (one after each semester); some universities ask for official language certificates for accepting credit students, while others just place a recommended language level; some require short-term students to have passed all their courses in previous academic years, while others accept students that have pending courses; etc.

5 As pointed by Wells (2014), apart from language and nationality, students make connections with people based on convenience and commonality of interests.

6 See, among others, (Dvir & Yemini, 2017; King & Ruiz-Celices, 2003; Lesjak et al., 2015; Llurda et al., 2016; Mitchell, 2012, 2015; Papatsiba, 2005, 2006; Parey & Waldinger, 2011; Powell & Finger 2013; Souto-Otero et al., 2013; Van Mol & Michielsen, 2015; Wilson 2011).

## Declarations

### Availability of data and material

List of reviewed articles available upon request. Not included as an annex in the final version of the article due to length requirements.
